# Renewable Polysaccharides as Supports for Palladium Phosphine Catalysts

**DOI:** 10.3390/polym10060659

**Published:** 2018-06-12

**Authors:** Oshrat Levy-Ontman, Shira Biton, Boris Shlomov, Adi Wolfson

**Affiliations:** Department of Chemical Engineering, Sami Shamoon College of Engineering, Basel/Bialik Sts., Beer-Sheva 8410001, Israel; shira.biton0@gmail.com (S.B.); boris.shlomov@gmail.com (B.S.); adiw@sce.ac.il (A.W.)

**Keywords:** catalysis, polysaccharides, heterogeneous catalysts, red algae, Suzuki cross-coupling

## Abstract

The investigation of the use of polysaccharides derived from natural sources to support metal catalysis has been the focus of several studies. Even though these molecules seem to be attractive materials, their full potential for use in support of heterogeneous catalysis still needs to be revealed. To that end, we developed a new preparation technique for polysaccharide-based palladium catalysts by immobilizing the palladium phosphine complexes on various renewable polysaccharides. The Suzuki cross-coupling in ethanol, using PdCl_2_(TPPTS)_2_ supported by various polysaccharides, was determined by gas chromatography and compared to homogeneous free-catalyst support. The PdCl_2_(TPPTS)_2_, that was immobilized on red algae supports, was successfully used as a heterogeneous catalyst in the Suzuki cross-coupling reaction, yielding high activity, higher than that of the homogeneous complex, without leaching. The FTIR spectrometry of representative heterogeneous polysaccharide-based TPPTS–PdCl_2_ catalysts was compared to that of native polysaccharide and polysaccharide-based TPP–PdCl_2_ catalysts, indicated on new bands, suggesting that the heterogenization occurs via interactions between the sulfonate group on the TPPTS and the hydroxyl groups on the polysaccharides. EDS and XPS analysis were also performed, confirming that the Pd complex was embedded within the *i*-carrageenan. A comparison of SEM images of *i*-carrageenan preparations also shed light on the interaction occurring between the polysaccharides and the TPPTS.

## 1. Introduction

Traditionally, fine and specialty chemicals were predominantly produced by means of a multistepped process of non-catalytic organic synthesis, yielding a large amount of organic waste [1]. Yet, over the past 50 years, much effort was devoted to the development of effective catalysts for organic synthesis that not only accelerate the reaction rate, but also do so in a more environmentally-friendly manner [2,3,4]. Moreover, the high industrial requirements for clean chemical production processes have increased the need for heterogeneous catalysts [4,5].

Currently, transition metal complexes (TMCs) are being extensively used as homogeneous catalysts in organic synthesis. Homogeneous catalytic methods are advantageous in that the reactions they enhance are simple, highly selective, and applicable to a variety of substrates. Modern organometallic chemistry has enabled the production of ideal catalysts that consist of the best matching of a metal with a ligand [1,2,3,4]. The great strength of homogeneous catalysis, especially when involving phosphine ligands, is the opportunity it provides for tailoring ligands, so as to enhance the reactivity and selectivity of metal-centered catalysts.

Since the 1960s, the heterogenization of homogeneous complexes has also been extensively studied [4,5]. This approach combines the advantages of heterogeneous catalysts (easy recycling, good stability) with those of their homogeneous counterparts (high activity and selectivity). The preparation of such heterogeneous catalysts requires some general considerations: (1) the preparation of the catalyst should be simple, efficient, and as generally applicable as possible; (2) the performance of the immobilized catalyst should be comparable to/or better than that of the free catalyst; (3) the separation of the heterogeneous catalyst from the reaction mixture, after the reaction, should be possible via simple filtration; (4) the leaching of the metal from the heterogenized catalyst should be minimal; (5) its reuse should be possible without a loss of activity; (6) the supports carrying the catalyst should be mechanically, thermally, and chemically stable; and (7) they should be compatible with the solvent and, preferably, commercially available in a good and reproducible quality.

Different organic and inorganic supports, such as polymers (e.g., polyvinyl alcohol and polydimethylsiloxane) and oxides (e.g., silica, alumina, zirconia and zeolite), were reported in the literature on TMCs being either immobilized by chemical modification and bonding, or entrapped or occluded [4,5]. As such, immobilization by means of a covalent bond usually requires tedious modification, and often leads to a loss of activity. Thus, the entrapment of a complex in a polymer matrix requires the careful choice of solvent, in order to achieve the appropriate swelling of the polymer while avoiding catalyst leaching.

Renewable and biodegradable organic polymers, biopolymers, and especially polysaccharides, have found multiple applications in various industries, such as pharmaceuticals, biomedical products, cosmetics and food, and also as fibers and building blocks in many engineered materials [6,7,8,9,10]. Due to their unique structures and functional groups, polysaccharides were also used as supports for metal catalysts. Moreover, the preparation of polysaccharide-supported catalysts for organic reactions, with or without modification of the support, has been gaining increased attention in the last decade; among them are starch [11], cellulose and hemicellulose [12,13,14,15,16], chitosan [17,18,19], guar-gum [20], carrageenans [21,22,23], and lignin [24,25]. Furthermore, these polysaccharides were also used as catalysts themselves. For instance, carrageenans, which have anionic sulfate groups in their structures, were used as heterogeneous Lewis acid catalysts [21,22], and *chitosan (C)*, that has amine groups in its structure, was used as a heterogeneous base in Michael additions [26]. Finally, polysaccharide matrices were also employed as supports for palladium nanoparticles and used in various catalytic transformations [27,28,29,30,31].

**Figure 1:**
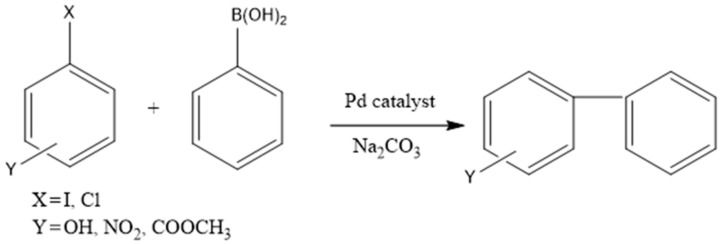
Suzuki cross-coupling of halobenzene and phenylboronic acid.

In this paper, we report the simple and efficient heterogenization of palladium phosphine complex on renewable polysaccharide supports. The heterogeneous catalysts were tested in the Suzuki cross-coupling of halobenzenes with phenylboronic acid, together with sodium carbonate as a co-catalyst, in ethanol [32,33,34,35] (Figure 1).

## 2. Materials and Methods

### 2.1. Polysaccharides and Reagents 

All polysaccharides and other chemicals (analytical grades) were purchased from Aldrich, Rehovot, Israel. The polysaccharide derived from *Porphyridium* sp. (*P*) was given as a gift for research purposes by Frutarom Ltd., Haifa, Israel.

### 2.2. Catalyst Preparation

A typical procedure for heterogeneous catalyst preparation was performed as follows: 10 µmol of palladium chloride were added to a vial with 3 mL distilled water, together with 30 µmol of a ligand (sodium triphenylphosphine trisulfonate, TPPTS), and mixed at room temperature for 5 min. Then, it was poured into a 15 mL polypropylene tube together with 3 mL of 1% *w*/*v* polysaccharide solution in distilled water, sealed, and mixed in a vortex for homogenization. When triphenylphosphine (TPP) was the ligand, it was first dissolved in 0.5 mL of ethanol, and then added to the aqueous solution of the polymer together with PdCl_2_. The next step was to deep freeze the tube at −20 °C for 24 h, until all the liquid was frozen. Then, the seal was removed, and the tube was covered with a paraffin sheet pierced by a disposable toothpick. The tube was then lyophilized for 48 h. At the end of this process, the dried “sponge-like” catalyst was cut into ~ 1 cm × 1 cm pieces and added to the reaction mixture.

### 2.3. Solubility 

The solubility of the lyophilized polysaccharide-based catalysts in the reaction mixture was compared to that of the native lyophilized polysaccharide (0.5% *w*/*v*). The lyophilized polysaccharides were prepared as detailed above, but without the catalyst supplement. Briefly, each one of the lyophilized samples was added to a vial with 5 mL ethanol, placed in a preheated oil bath at 60 °C, and magnetically stirred for 24 h. At the end of this process, the solubility of the support was assessed. If the polysaccharide kept its “sponge-like” texture and the mixture was clear, it was termed “immiscible”; otherwise, it was termed “miscible”.

### 2.4. Reaction Procedure

In a typical procedure, 10 µmol of palladium chloride with 30 µmol of the ligand (homogenous or heterogeneous) were added to a vial with 5 mL solvent, together with 0.5 mmol halobenzene, 0.75 mmol phenylboronic acid, and 0.6 mmol Na_2_CO_3_. The mixture was placed in a preheated oil bath at 60 °C, and magnetically stirred for 24 h. At the end of the reaction, the reaction mixture was cooled. In cases in which a heterogeneous catalyst was used, it was removed by filtration. Then, the organic phase was analyzed to determine the reaction conversion by gas chromatography (GC), using a HP-5 column. Finally, the turnover frequency (TOF) of the reaction was calculated as follows: TOF = conversion × (0.5 × 10^−3^/(10 × 10^−6^ × 24)). The average sum-square peak ratios were calculated according to a calibrated reactant–product curve. 

### 2.5. Leaching Analysis

Leaching of the catalysts was tested in three ways: (1) performing a second reaction after the removal of the catalyst from the original reaction mixture, and the addition of the catalyst to a fresh reaction mixture with the corresponding amounts of fresh substrates and sodium carbonate, then checking if the catalytic performance was comparable; (2) proceeding with the reaction after the removal of the catalyst by running the reaction mixture under similar conditions for an additional 24 h, to check if the conversion increases with time; and (3) by doing an inductively-coupled plasma optical emission spectrometry (ICP-OES) (Arcos, Spectro) analysis of the reaction medium after a 24 h reaction time and the removal of the catalyst, to check for palladium leftover in the solution. 

### 2.6. Catalyst Recycling

Catalyst recycling was performed by the addition of the recovered catalyst to a solution with similar amounts of fresh substrates and base, and running the reaction mixture under similar reaction conditions for an additional 24 h.

### 2.7. FTIR Analysis

A Fourier-transform infrared (FTIR) analysis of lyophilized polysaccharides was performed using a FTIR Nicolet 6700 (Thermo Scientific, Waltham, MA, USA) spectrophotometer with an attenuated total reflectance (ATR) accessory with a diamond crystal. The recorded spectra were the means of 36 spectra taken in the 650–4000  cm^−1^ range with a 0.5  cm^−1^ resolution, and atmospheric correction switched on at room temperature (25 °C).

### 2.8. Energy Dispersive X-Ray Spectrometry (EDS)

Elemental analysis of *i*-PdCl_2_(TPPTS)_2_ was performed using scanning electron microscope (SEM), FEI Verios 460L XHR (extreme high resolution, Hillsboro, OR, USA), equipped with energy-dispersive X-ray spectroscopy (EDS, Oxford Instruments, Oxford, UK).

### 2.9. Surface Analysis by X-ray Photoelectron Spectroscopy (XPS) 

XPS data were collected using an X-ray photoelectron spectrometer ESCALAB 250 ultrahigh vacuum (1 × 10^−9^ bar) apparatus with an AlK^α^ X-ray source and a monochromator. The X-ray beam size was 500 μm and survey spectra was recorded with pass energy (PE) 150 eV, and high energy resolution spectra were recorded with pass energy (PE) 20 eV. To correct for charging effects, all spectra were calibrated relative to a carbon C 1s peak positioned at 284.8 eV. Processing of the XPS results was carried out using AVANTGE program.

### 2.10. SEM Analysis

Lyophilized polysaccharide samples were previously coated with gold. Scanning electronic microscopy (SEM) was performed using Quanta 200, FEI (Thermo-Fisher, Waltham, MA, USA). Acceleration voltage was 25 kW.

### 2.11. High-Resolution Transmission Electron Microscopic (HRTEM) Analysis

High-resolution transmission electron microscopic (HRTEM) micrographs were obtained using an EFI Talos F200C transmission electron microscope (TEM) operated at 200 kV at room temperature. The samples were prepared by the deposition of a drop of ethanol suspension of the crushed solid catalyst on a carbon-coated Cu grid.

## 3. Results

**Figure 2:**
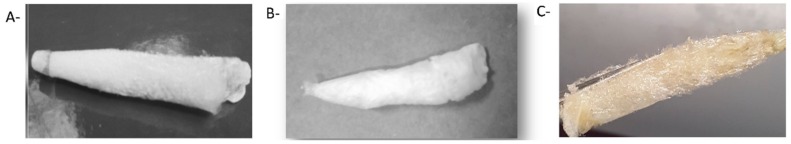
Lyophilized *i*-carrageenan: (**A**) native polysaccharide (free-catalyst support); (**B**) PdCl_2_(TPP)_2_ supported on polysaccharides; (**C**) PdCl_2_(TPPTS)_2_ supported on polysaccharides.

The first step towards yielding a heterogeneous system is to make sure that the support will not dissolve in the reaction mixture. Suzuki cross-coupling reactions are traditionally performed in polar solvents, like dimethylformamide (DMF) and dimethyl sulfoxide (DMSO), and various alcohols, since it involves polar and apolar organic substrates, an inorganic base, and a metal catalyst. Thus, ethanol, which is polar, but a more environmentally-friendly solvent, was used as the solvent of choice. The investigation began by studying the solubility of various polysaccharides in ethanol under Suzuki reaction conditions (Table 1). Different types of commercial natural polysaccharides were tested: red macroalgae carrageenans—*iota* (*i*), *kappa* (*κ*), and *lambda* (λ), that have hydroxyl and anionic sulfate groups in their structures; red microalgae polysaccharide (*P*), that have hydroxyl and anionic carboxyl and sulfate groups (~9%) in their structures; *chitosan* (*C*), that has hydroxyl and amine groups in its structure; *xanthan gum* (*X*), that has hydroxyl, pyruvate, and carboxylate groups; and *guar* (*G*) and *locust bean* (*LB*) gums, that have only hydroxyl groups in their structures. All the lyophilized polysaccharides yielded a “sponge-like” texture. A representative illustration of lyophilized *i* is shown in Figure 2A.

As illustrated in Table 1, the two-branched polysaccharides with only hydroxyl groups, *G* and *LB*, fully dissolved in ethanol, while all the other polymers that have other functional groups, besides hydroxyl, did not dissolve in ethanol (i.e., the sulfate (*i*, *k*, λ and *P*) and amine (*C*) groups, pyruvate (*X*), and carboxylate (*X*, *P*)). The solubility of various polysaccharides in solution is dependent on certain parameters, including solvent type, polysaccharide concentration, and the nature of the polysaccharide (e.g., molecular weight, composition, functional groups, quantity, and three-dimensional (3D) structure) [36,37,38]. In general, most polysaccharides are far less soluble in water or other protic and polar solvents than their monomers, due to polymer linkage between the sugar’s reactive groups, preventing them from interacting with water. In addition, steric hindrance in the formed polymer may also decrease the ability of adjacent hydroxyl groups to interact with water. Lastly, hydroxyl groups may interact on a polymer backbone via intra- and intermolecular hydrogen bonds, when amine groups are added to the sugar structure and an amide bond is formed, or when sulfate salts are present that interact with the hydroxyl group to form sulfate esters. Thus, it seems that, although hydrogen bonds between the hydroxyl groups of all polysaccharides may lead to the formation of intermolecular bonding that reduces their solubility in ethanol, the low concentrations of the polysaccharide in ethanol (0.3% wt) enables the solubility of *G* and *LB*. Nonetheless, the existence of other functional groups on the polymer backbone led to the formation of stronger chemical bonds (i.e., amide and sulfate ester, correspondingly), thus resulting in immiscibility, even in ethanol. 

The second stage in the study was to add palladium catalyst to the polysaccharides and prepare heterogeneous analogues. Various palladium salts and complexes were reported to be suitable catalysts for Suzuki synthesis, whereas simple and commercially-available ligand-like TPP is often employed. Thus, PdCl_2_(TPP)_2_ was the first catalyst chosen. Furthermore, the water-soluble derivative of TPP, TPPTS (also known as 3,3′,3″-phosphanetriyltris (benzenesulfonic acid) trisodium salt) was also tested, since it was assumed that the TPPTS, which has sulfonate groups, might interact with the hydroxyl groups on the polysaccharide [21]. 

Note that the catalyst-free *i*-support (Figure 2A) and the *i*-PdCl_2_(TPP)_2_ catalyst (Figure 2B) both had similar textures, while the *i*-PdCl_2_(TPPTS)_2_ catalyst (Figure 2C) was much more porous, tending to be easily broken under external stress. This observation suggested that the use of TPPTS as a ligand might yield new interactions.

**Figure 3:**
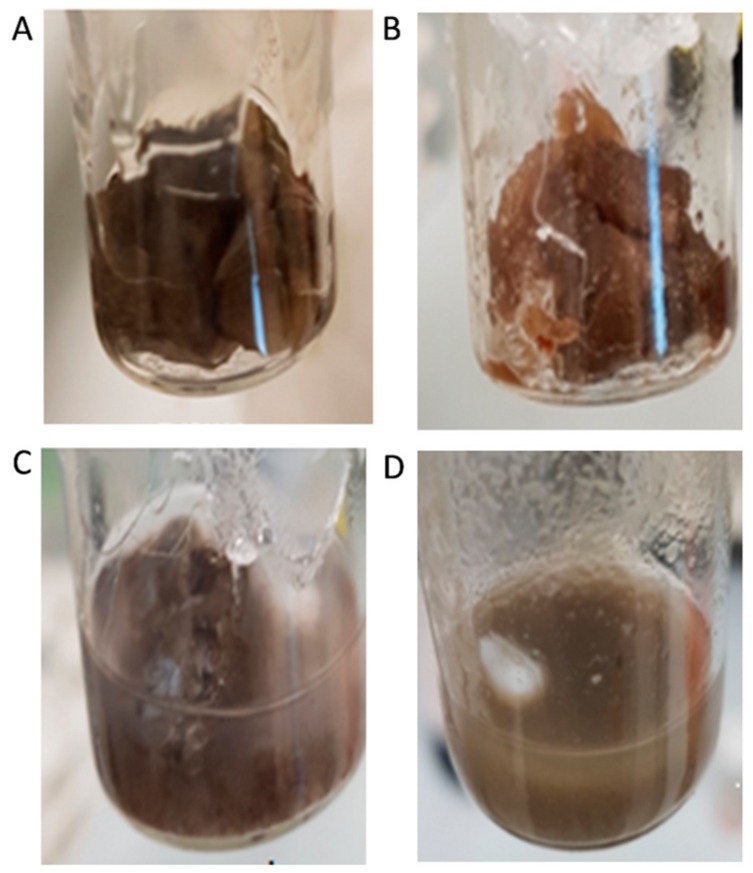
Red-algae polysaccharide-based PdCl_2_(TPPTS)_2_ catalysts after 24 h at 60 °C in ethanol: (**A**) *i*; (**B**) *κ;* (**C**) *λ*; (**D**) *P*.

The addition of various polysaccharide-based PdCl_2_(TPP)_2_ catalysts to ethanol under reaction conditions (60 °C, 24 h) yielded a dark-colored solution, indicating that the catalyst had leached out of the support. However, the mixing of the various supported PdCl_2_(TPPTS)_2_ catalysts in ethanol under reaction conditions, did not show any dark coloration, even after 24 h. As with the catalyst-free supports, PdCl_2_(TPP)_2_ and PdCl_2_(TPPTS)_2_, supported on *X*, *G*, and *LB*, were fully dissolved in ethanol, while PdCl_2_(TPP)_2_ and PdCl_2_(TPPTS)_2_, supported on red-algae polysaccharides (*i*, *κ*, λ and *P*), did not dissolve (Table 1). As previously mentioned, the immiscibility properties of the various red-algae polysaccharide supports, in ethanol under the reaction conditions, may be attributed to their unique composition (comprised of sulfate ester groups that are not found in the other tested polysaccharides) and their 3D structures. Yet, in some of the preparations, the addition of PdCl_2_(TPPTS)_2_ to the polysaccharides yielded more crumbled solid than compared with catalyst-free support (Figure 3). For example, PdCl_2_(TPPTS)_2_ supported on *i* and *κ* became stiff in ethanol (Figure 3A,B, respectively), while the *λ*-based catalyst stayed softer (Figure 3C), and the *P*-based catalyst became powder-like (Figure 3D).

The solubility of the carrageenans in water and protic solvents, like ethanol, depends on their structures and, mainly, on the balance between hydrophilicity, as provided by the hydroxyl group, and hydrophobicity, due to the 3,6-anhydro-D-galactose residues. As such, *λ*, which is not composed of the 3,6-anhydro-D-galactose group in each repeating unit (as in the other carrageenans), yielded softer catalyst support in ethanol [38]. Contrarily, *i* and *κ*, with only one 3,6-anhydro-D-galactose group in each repeating unit, yielded the stiffest catalyst-support texture. Finally, the powder-like texture of *P*, may be explained by its heteropolymeric structure. Yet, the structure of *P* is not fully resolved; it is composed of aldobiouronic acid 3-*O*-(-d-glucopyranosyluronic acid)-l-galactopyranose disaccharide, which was also part of a larger linear building block, containing (1 → 2 or 1 → 4)-linked xylopyranosyl, (1 → 3)-linked galactopyranosyl, and (1 → 3)-linked glucopyranosyl or glucopyranosyluronic acid residues [39,40]. Solutions of the sulfated polysaccharides of *Porphyridium* sp. tend to be highly viscous at low polymer concentration, and to be stable at a wide range of pH values, temperatures, and salinities. It was suggested that the *P* chains in solution adopt a helical, wormlike chain cylinder model consisting of helical segments (with a pitch of 1.6 nm) joined by more flexible moieties [39,40,41]. 

The Suzuki reactions with the various PdCl_2_(TPPTS)_2_-supported polysaccharide catalysts were tested in ethanol using iodobenzene and phenyboromic acid as representative substrates (Figure 1), and the results are summarized in Table 2. Surprisingly, employing the four heterogeneous systems, using *i*, *κ, λ* and *P* as supports (Table 2, entries 2–5) yielded higher performance than the corresponding homogeneous reaction (Table 2, entry 1). In order to be sure that the complex had not leached out of the supports, the catalysts were removed from the reaction mixtures at the end of the reaction; then, the reaction proceeded to run under similar conditions for an additional 24 h. It was found that the TOF did not increase over time, indicating that there were no catalysts left in the solution. In addition, an ICP-OES analysis of the reaction mixture after a 24 h reaction, the removal of the catalyst was also executed, and no palladium leftovers were found in the solution. Thus, it was proven that the new system is indeed heterogeneous. 

The increased TOF in the presence of the polysaccharides proved that they had accelerated the reaction. Furthermore, creating this reaction with the various soluble polysaccharides (entries 7–9), except for *C* (entry 6), also resulted in its acceleration, compared to a homogeneous reaction (entry 1). Moreover, when the supports dissolved in the reaction mixture (entries 7–9), the acceleration of the reactions, compared to those of the homogeneous system, were even higher, as expressed by the higher TOFs, probably since the heterogeneous system still has a certain mass-transfer limitation. It might be that the hydroxyl groups on the polymer backbone are the main cause of the higher performance; several previous works reported that addition of water to a Suzuki cross-coupling reaction mixture of isopropanol [42] or tetrahydrofuran (THF) [43] provided more polar and protic conditions and, thereby, produced higher activity. Moreover, it was found that water accelerated the Pd-catalyzed amidation of *o*-bromotoluene in various organic solvents, probably because it increased the solubility of the base acting as co-catalyst [44]. As such, it may be that the high concentration of hydroxyl groups in the polymer structure served as water, and enabled the higher solubility of both the phenylboronic acid and the potassium carbonate in the reaction mixture, leading to higher yields. Indeed, the addition of 2 mL of distilled water to the homogeneous reaction in ethanol also resulted in acceleration of the reaction (entry 11). Another possible acceleration mechanism may be the stabilization of the complex by ligand–polysaccharide interactions, thus avoiding its deactivation due to the formation of palladium black by oxidation. Indeed, it was already reported that performing the Suzuki reaction in ethanol involves the generation of palladium black nanoparticles, which reduces the catalyst activity [45]. Lastly, though the TOF with *C* (entry 6), which yields heterogeneous catalysts, was very low, the addition of catalyst-free solid *C* to the homogeneous reaction (entry 10) resulted in increased TOF, compared to conventional homogeneous reactions (entry 1), indicating that, like all other polysaccharides, *C* itself also accelerates the reaction. Thus, the low TOF with the heterogeneous *C* catalyst may be attributed either to the lower mass transport via the support (i.e., being denser than carrageenan-based supports) or to the deactivation of the catalysts, due to the interaction of the palladium center with the amine groups of the chitosan.

To better evaluate the applied potential of the new heterogeneous system, PdCl_2_(TPPTS)_2_, supported on *I*, was recycled 7 times (Table 3). As illustrated in Table 3, these catalysts may be recycled with only minor loss of activity. Since the TOF values of the reactions did not increase after the removal of the catalyst, and no palladium was observed in the solution by ICP-OES detection, the conversion loss may be attributed either to the deactivation of the complex or to the loss of some catalyst during the separation step.

To study the scope of the new *i*-heterogeneous system, the catalyst was employed using different halobenzenes, as illustrated in Table 4.

As expected, replacing the iodobenzene (entry 1) with chlorobenzene (entry 2) resulted in a lower conversion value [46]. Yet, the addition of an electron-donating group to the chlorobenzene, as in the case of the 4-chlorobenzyl alcohol (entry 3), activated the aromatic ring and allowed better release of the chloro-substituent. Contrarily, the addition of electron-withdrawing groups, like in the cases of the 4-chloroacetophenone (entry 4) and the 1-chloro-3-nitrobenzene (entry 5), yielded lower conversion values than in the conversion done with chlorobenzene [47]. Finally, in some reactions, the activity of the heterogeneous system was higher than that of the homogeneous system.

The final stage of this study was to characterize the new heterogeneous catalysts, to learn about the interaction between the complex and the polysaccharide, and to gain knowledge of the structure of the new composite. The characterization was performed with PdCl_2_(TPPTS)_2_ supported on *i*, using FTIR, SEM-EDS, XPS, SEM, and TEM analyses.

**Figure 4:**
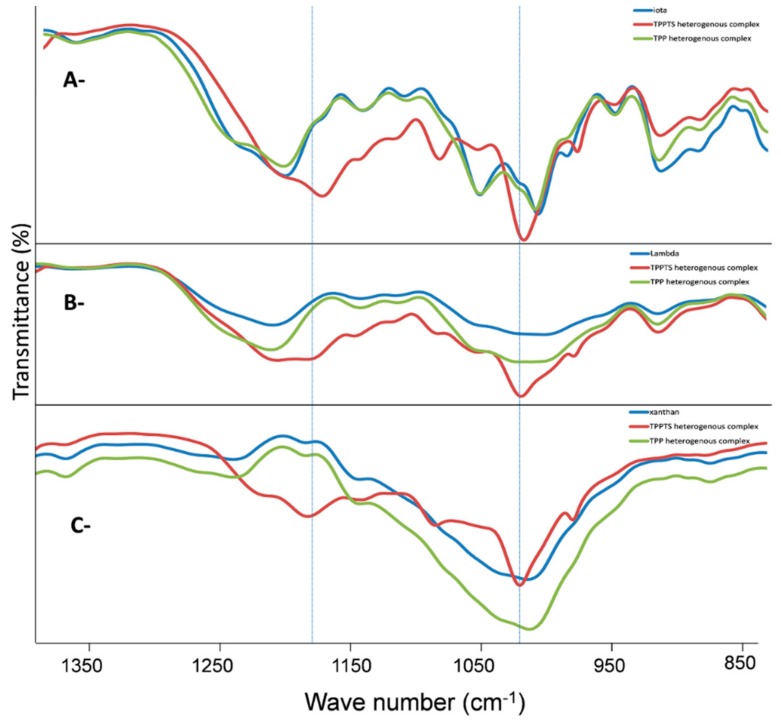
Infrared spectra of native polysaccharide and polysaccharide with PdCl_2_(TPP)_2_ and PdCl_2_(TPPTS)_2_: (**A**) *i*, (**B**) *λ*, (**C**) *X*.

In order to confirm the hypothesis that the Pd catalyst does not leach into the reaction mixture due to the chemical linkage between the TPPTS and the polysaccharide, the FTIR spectra of three samples: native-*i*, *i*-PdCl_2_(TPP)_2_, and *i*-PdCl_2_(TPPTS)_2_, were compared. As demonstrated in Figure 4A, the spectra of native-*i*, and *i*-PdCl_2_(TPP)_2_ were similar, and showed comparable behavior to published data about *i*, having characteristic bands [48,49]. However, the FTIR of *i*-PdCl_2_(TPPTS)_2_ yielded two additional bands (1030, 1170 cm^−1^), which were not present in the other spectra. These bands may be attributed to sulfonate ester, C–O–S, which may be formed by interactions between the sulfonate groups of the TPPTS and the hydroxyl groups of *i* or, less likely, with the 3,6 D-anhydrogalactose. Since the 1070 cm^−1^ peak, an indication of 3,6 D-anhydrogalactose [49], is seen in all the samples, the sulfonate ester linkage is probably formed with the free hydroxyl groups of the other C-positions. To bolster the finding that the sulfonate ester is being formed by the hydroxyl group of the polysaccharide and the sulfonate group of the TPPTS, the FTIR spectra of both the *λ*- and the *X*-PdCl_2_(TPPTS)_2_, which have no sulfate groups, were also analyzed, yielding similar peaks as with *i*-PdCl_2_(TPPTS)_2_ (Figure 4B,C, respectively). This observation demonstrates that, indeed, the new bond is formed between the sulfonate groups of TPPTS and the hydroxyl group of the polysaccharide.

**Figure 5:**
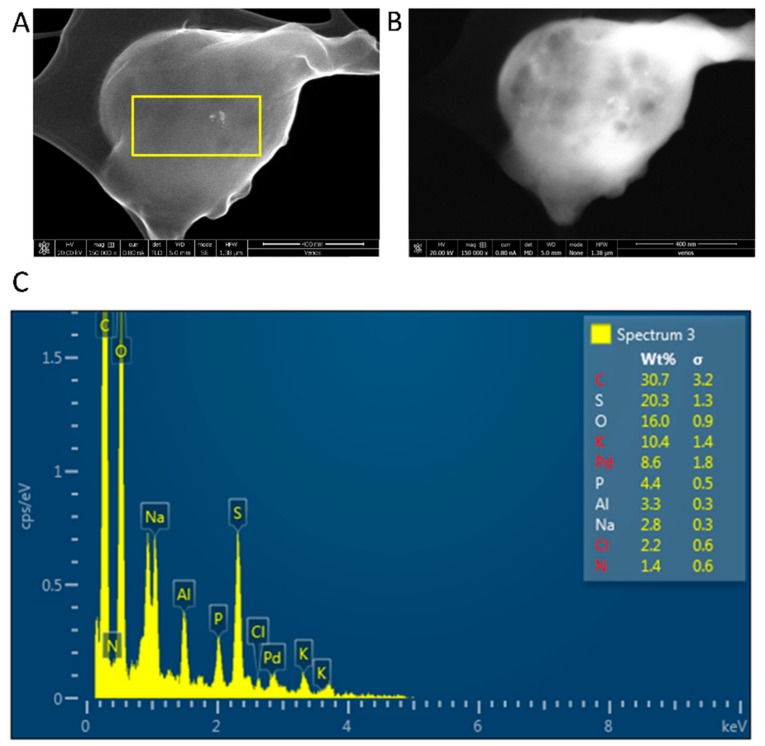
SEM-EDS analysis of PdCl_2_(TPPTS)_2_ supported on *i*: (**A**) secondary electron imaging; (**B**) backscattered electron imaging; (**C**) EDS spectrum and tabulated results of EDS. The yellow rectangle in the inset image in Figure 5A shows the selected EDS inspection field.

An elemental analysis, using SEM-EDS of the *i*-PdCl_2_(TPPTS)_2_, was also conducted. A representative EDS spectrum revealed that all the expected elements, that were involved in the heterogeneous catalyst preparation, are also observed in the final sponge-like structure (Figure 5). The detectable amounts of Pd and P elements, derived from the PdCl_2_(TPPTS)_2_ complex, are noteworthy. One of the prominent findings is that the molar-elemental ratio of P:Pd in the heterogeneous catalyst is 2:1, as it is in the homogeneous catalyst. Moreover, the scans of various samples yielded the same P:Pd molar-elemental ratio (data not shown). Therefore, it may be suggested that the complex (PdCl_2_(TPPTS)_2_) kept its chemical structure.

Surface-sensitive technique, XPS, was also applied to analyze the surface composition and electronic structure of the *i* and the *i*-PdCl_2_(TPPTS)_2_ sponges. The XPS survey of *i*-PdCl_2_(TPPTS)_2_ shows the presence of Pd, Cl, and P elements, in contrast to that of *i* (Table 5). Indeed, the presence of these elements agrees with the EDS results, demonstrating that the PdCl_2_(TPPTS)_2_ complex is embedded within the heterogeneous catalyst. 

**Figure 6:**
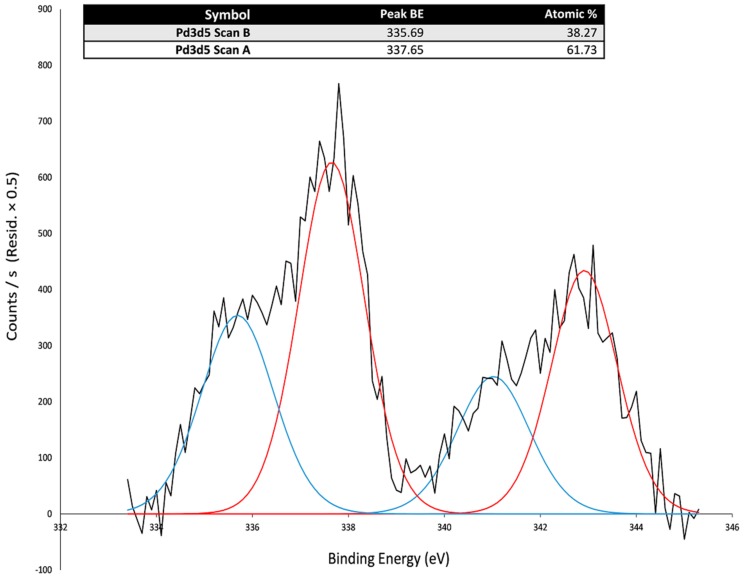
XPS spectra of *i*-PdCl_2_(TPPTS)_2_ in the Pd3d region.

A deeper analysis of the Pd (Pd3d peak) is presented in Figure 6. These spectra show two peaks in the energy range from 334 to 346 eV. Figure 6 reveals that Pd exists in at least two different forms. It also suggests that 38.27% of Pd(II), derived from the PdCl_2_, is reduced to a metallic form, with the binding energies of Pd(0)3d (d5 335.69 eV; d3 340.59 eV, Figure 6). 

**Figure 7:**
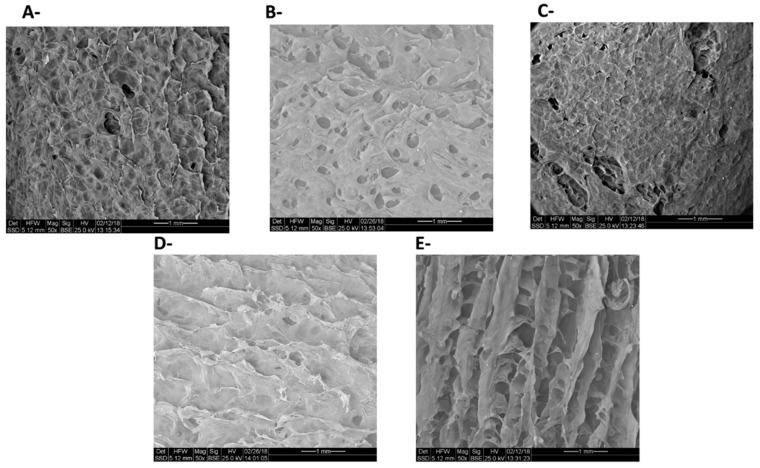
SEM micrographs of (**A**) *i*; (**B**) *i*-PdCl_2_; (**C**) i-TPP; (**D**) *i*-TPPTS; (**E**) *i*-PdCl_2_(TPPTS)_2_.

To further investigate the heterogeneous catalyst structure, SEM analysis was also done (Figure 7). Firstly, the SEM image of native *i* was compared with the image of *i*-PdCl_2_; while the image of the native *i* was characterized by irregularly-shaped particles with laminar surfaces (Figure 7A), that of the *i*-PdCl_2_ was characterized by smooth, porous surfaces (Figure 7B). In general, it is well known that addition of cations, such as, K^+^, Ca^2+^, Co^2+^, and Fe^+3^ to the anionic sulfate groups in *i*-carrageenan aqueous solution can produce sol–gel by the formation of a “double-helix” structure [50]. In that case, the cations interact with the sulfate groups by means of ionic forces [51,52] and assist in the formation of the “double-helix” structure, but they may also promote the aggregation of different “double-helix” structures to form junction zones [53]. In addition, the cations can also interact with the hydroxyl groups on the carrageenan structure, as previously proposed regarding different metal cations supported on carrageenans [21,54]. Hence, it may be suggested that pallidum ions might also interact with the carrageenans, yielding a less ordered packing of each single chain. The addition of TPP to *i* (Figure 7C) yielded an image similar to that of the native *i* (Figure 7A). Yet, the addition of TPPTS to *i* (Figure 7D) also resulted in non-smooth surfaces with channels (Figure 7E), again implying an interaction between the TPPTS and the polysaccharide. Finally, the *i*-PdCl_2_(TPPTS)_2_ yielded a reticular porous arrangement (Figure 7F). This may perhaps be attributed to the fact that PdCl_2_(TPPTS)_2_ has two TPPTS ligands, yielding a larger, unique structure. Indeed, PdCl_2_(TPPTS)_2_ may be formed both by cis and trans isomers; whereas the cis isomer is more common, and both distinct compound configurations have planar structures [55]. This means that the complex is bigger than either the PdCl_2_ or the TPPTS alone, and that the angle between the two TPPTS ligands is around 90°. Hence, the polysaccharide chains may be arranged around the complex, interacting with the six sulfonate groups of TPPTS, only in defined directions in the open space. 

**Figure 8:**
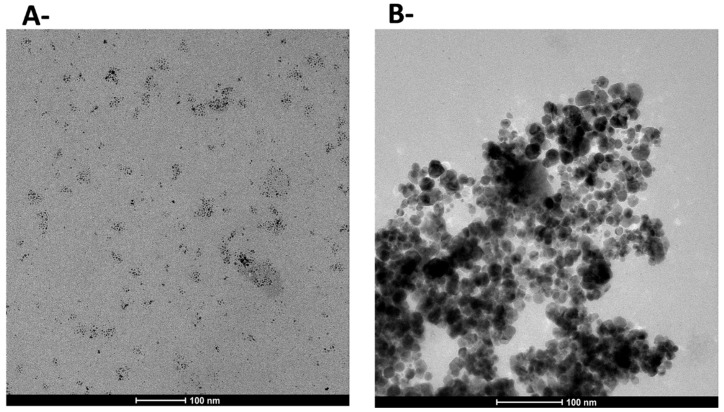
TEM micrographs of (**A**) fresh i-PdCl_2_(TPPTS)_2_ and (**B**) i-PdCl_2_(TPPTS)_2_ after the reaction.

Finally, the heterogeneous catalyst that turned black after being used in the reaction (Figure 3), indicated that Pd nanoparticles are formed while heating the catalyst under reaction conditions [12,13,14,15]. Thus, TEM analyses were performed of both the fresh catalyst and of the catalyst after the reaction (Figure 8). The TEM image of the heterogeneous catalyst, *i*-PdCl_2_(TPPTS)_2_ (Figure 7A), shows that the complex was imbedded and suspended in the polysaccharide matrix, yielding small nanoparticles that were created by the lyophilization of the water-soluble complex and the precipitation of the complex in the polysaccharide matrix. However, employing the catalysts in the reaction, under 60 °C for 24 h, resulted in the formation of much bigger nanoparticles, as illustrated in Figure 7B. This implies that these reaction conditions promote the aggregation of nanoparticles, as well as the reduction of the complex [44]. Moreover, these newly-formed nanoparticles may explain the slight reduction in catalyst activity during the recycling cycles (Table 3).

## 4. Conclusions

Performing Suzuki cross-coupling in the presence of PdCl_2_(TPPTS)_2_, supported on various polysaccharides in the new preparation we developed, revealed that polysaccharides accelerate the reaction, as compared to that reaction performed with a homogenous complex and without polysaccharides. Comparative FTIR analyses of various polysaccharides containing TPP, TPPTS, and native polysaccharide, suggested the possibility of new sulfate ester bonds, formed between the sulfonate groups of the TPPTS and the hydroxyl groups of the polysaccharides. Among the various polysaccharides tested, only those derived from red algae were successfully heterogenized and yielded high conversion values.

The *i*-carrageenan was successfully used to immobilize PdCl_2_(TPPTS)_2_, and was efficiently reused seven times as a heterogeneous catalyst in Suzuki cross-coupling in ethanol. It was found that only the TPPTS-based complexes were attached to the support, and did not leach into the reaction mixture.

## Figures and Tables

**Table 1:** Miscibility of polysaccharides in ethanol ^a^.

Polysaccharide	Functional Groups	Branched/Linear	Building Block	Miscibility in Ethanol
*i*	–OH,–OSO_3_^−^	Linear	d-Gal-4-sulfate,3,6-anhydro-d-Gal-2-sulfate	NoNo ^b^
*κ*	–OH,–OSO_3_^−^	Linear	d-Gal-4-sulfate,3,6-anhydro-d-Gal	NoNo ^b^
λ	–OH,–OSO_3_^−^	Linear	d-Gal-2-sulfate, d-Gal-2,6 disulfate	NoNo ^b^
*P*	–OH,–OSO_3_^−^–COO^−^	Branched	Not defined	NoNo ^b^
*C*	–OH,–NH_2_	Linear	β-(1 → 4)-linked d-glucosamine and *N*-acetyl-d-glucosamine	NoNo ^b^
*X*	–OH–CH_2_OCOCH_3_–COO^−^	Branched	β-(1 → 4)-d-glucopyranose	NoYes ^b^
*G*	–OH	Branched	β-(1 → 4)-linked mannose	YesYes ^b^
*LB*	–OH	Branched	β-(1 → 4)-linked mannose	YesYes ^b^

^a^ 0.03 g of polysaccharide, 60 °C, 24 h, 5 mL ethanol. ^b^ Addition of 10 μmol PdCl_2_(TPPTS)_2_.

**Table 2:** Suzuki cross-coupling in the presence of PdCl_2_(TPPTS)_2_, supported on various polysaccharides ^a^.

Entry	Polysaccharide	TOF (h^−1^)
1	Non	0.846
2	*i*	1.319
3	*κ*	1.621
4	*λ*	1.150
5	*P*	1.319
6	*C*	0.119
7	*G*	2.073
8	*X*	2.063
9	*LB*	2.077
10	*C* ^b^	2.056
11	Non ^c^	2.065

^a^ Reaction conditions: 0.5 mmol iodobenzene, 0.75 mmol phenylboronic acid, 10 µmol catalyst, 0.6 mmol Na_2_CO_3_, 5 mL ethanol, 60 °C, 24 h. ^b^ Addition of lyophilized chitosan that was prepared without a catalyst to the reaction mixture. ^c^ Addition of 2 mL distilled water.

**Table 3:** Recycling of PdCl_2_(TPPTS)_2_ supported on *I* ^a^.

Entry/Cycle	TOF (h^−1^)^b^
1	1.063
2	0.966
3	0.955
4	0.900
5	0.863
6	0.722
7	0.700

^a^ Reaction conditions: 0.5 mmol iodobenzene, 0.75 mmol phenylboronic acid, 10 µmol catalyst, 0.6 mmol Na_2_CO_3_, 5 mL ethanol, 60 °C, 24 h.

**Table 4:** Heterogeneous reactions with representative substrates ^a^.

Entry	Halobenzene	Homogeneous TOF (h^−1^)	Heterogeneous TOF (h^−1^) ^b^
1	Iodobenzene	0.846	1.319
2	Chlorobenzene	0.538	0.440
3	4-Chlorobenzyl alcohol	1.098	1.283
4	4-Chloroacetophenone	0.479	0.313
5	1-Chloro-3-nitrobenzene	0.106	0.223

^a^ Reaction conditions: 0.5 mmol halobenzene, 0.75 mmol phenylboronic acid, 10 µmol PdCl_2_(TPPTS)_2_, 0.6 mmol Na_2_CO_3_, 5 mL solvent, 50 °C, 24 h. ^b^ Using *i* as a support.

**Table 5:** Elemental identification and quantification of *i* and *i*-PdCl_2_(TPPTS)_2_ determined by XPS.

	*i*		*i*-PdCl_2_(TPPTS)_2_	
Name	Peak BE (eV)	Atomic %	Peak BE (eV)	Atomic %
P2p	-	-	130.13	1.09
S2p	166.97	5.55	166.58	7.96
Cl2s	-	-	266.22	0.78
C1s	283.11	51.51	282.85	47.87
Pd3d	-	-	334.27	0.30
K2s	-	-	375.55	0.79
N1s	396.93	3.37	397.46	1.86
Ca2s	345.19	2.34	436.79	0.81
O1s	529.89	36.53	529.64	35.41
Na1s	1070.56	0.70	1069.79	3.13
